# Editorial: Wearable biosensing devices

**DOI:** 10.3389/fbioe.2025.1574786

**Published:** 2025-03-26

**Authors:** Faxing Zou, Tailin Xu

**Affiliations:** The Institute for Advanced Study (IAS) Shenzhen University Shenzhen, Shenzhen, Guangdong, China

**Keywords:** wearables, bioelectronics, electrochemistry, biosensors, portable devices, myoelectric control, biological signals

## Introduction

In recent years, the rapid development of wearable bio-intelligent sensing devices and signal processing algorithms has opened new paradigms in healthcare management. Electrical sensors and wearable devices, with their high sensitivity and real-time monitoring capabilities, have demonstrated significant advantages in chronic wound care and biomarker monitoring, thereby providing robust support for early disease diagnosis and precision treatment. Concurrently, big data models, through the integration of extensive clinical datasets, have driven intelligent upgrades in neuroregulation and prosthetic control systems, enabling the formulation of more refined individualized rehabilitation plans. Notably, recent advancements in biosensing technologies, wearable devices, big data analytics, and adaptive algorithms have collectively established both theoretical foundations and practical frameworks for constructing efficient, safe, and intelligent healthcare management systems.

This Research Topic comprises six papers, including four original research articles, one review, and one perspective article. These studies concentrate on key domains such as chronic wound care, biomarker monitoring, neuroregulation, and prosthetic control, and explore the latest advancements in electrochemical sensing, wearable devices, big data analytics, and adaptive algorithms, while also examining their potential clinical applications.

## Wound care

Chronic wounds resulting from injuries, surgical procedures, or underlying diseases present substantial clinical challenges and impose a heavy economic burden. There is an urgent need for effective interventions to mitigate severe complications associated with these conditions. Li et al. provide a comprehensive overview of closed-loop wound management systems, emphasizing the integration of wound biomarker detection, assessment, and on-demand treatment through flexible electrochemical devices. The authors further analyze challenges, opportunities, and future directions for soft and stretchable electrochemical biosensors, with the goal of improving the efficiency and timeliness of wound care.

## Biomarker monitoring

Due to the complex conditions of open wound environments, electrochemical biosensors still face persistent challenges in stability and specificity. Building on this research, the ELSAH (electronic smart patch system for wireless monitoring of molecular biomarkers for healthcare and wellbeing) smart patch project has developed a microneedle-based platform for continuous monitoring of multiple biomarkers (Brinkmann et al.). This innovative platform employs wireless technology to enable real-time analysis of glucose and lactate levels, thereby advancing early diabetes detection and personalized health management. Recently, wearable biosensing devices have been applied in neurosurgery. For example, Zarrin et al. developed a novel method for accurately measuring cerebrospinal fluid (CSF) flow rates through VP shunts. This approach uses an inductive principle-based VP shunt flow sensor to monitor CSF flow by detecting changes in electrical charge density, achieving low power consumption (37.5 μJ per measurement) and high precision (error <5%). This advancement offers a reliable tool for the long-term outpatient management of hydrocephalus patients.

## Neuroregulation and prosthetic control

Similarly, with the rapid development of surface electromyography (sEMG) in the field of biomedical engineering, wearable biosensing devices capable of accurately reflecting changes in the nervous and muscular systems have also been developed. Recent advancements in surface electromyography (sEMG) signal processing have significantly enhanced the accuracy and reliability of myoelectric control systems. Lu et al. introduced an improved multi-layer wavelet transform algorithm to preprocess raw sEMG signals, effectively removing background noise and power frequency interference. Subsequently, an enhanced Fast-ICA algorithm was used to separate denoised signals, enabling the elimination of electrocardiogram (ECG) artifacts from upper limb sEMG recordings. Notably, Eddy et al. demonstrated that large multi-user models could achieve a 93.0% classification accuracy for six gestures in a cohort of 306 unseen users. By adopting a discrete approach to classification—recognizing entire dynamic gestures as single events—the study underscored the potential of big data approaches in enabling robust cross-user myoelectric control. Furthermore, it is crucial to eliminate artifacts during the use of wearable biosensing devices, Lee et al. developed an adaptive artifact cancellation filter to address artifacts caused by transcutaneous electrical nerve stimulation (TENS) feedback in sEMG signals during the use of prosthetic hand. This method utilizes a modified least-mean-square adaptive filter that references the mean of previous artifacts and compensates using prior information of the TENS system. The approach has been validated in prosthetic hand control, showing significant improvements in signal quality and intention estimation. This will enrich the functionality of prostheses and bring great convenience to the lives of prosthetic users.

## Perspectives and conclusion

The collection of six papers in the Research Topic represents significant progress in chronic wound care, biomarker monitoring, neuroregulation and prosthetic control. These studies concentrate on these key domains, have played a significant role in advancing the fields of electrochemical sensing, wearable devices, big data analytics, and adaptive algorithms. It not only highlights the potential clinical applications but also offers promising avenues for future investigations.

In conclusion, this study presents the latest advancements and applications of wearable biosensing devices. We aim to provide readers with a timely and engaging overview that inspires future research in biotechnology, wearable sensing, and interdisciplinary studies in life and health sciences. This effort seeks to promote the integration of wearable biosensing technologies and the development of intelligent healthcare systems.

